# Effect of Essential Oil Addition on PLA/PBAT Blend Properties for Biodegradable Packaging Applications

**DOI:** 10.1021/acsomega.5c10845

**Published:** 2026-03-23

**Authors:** Murilo B. Valério, Ana Lúcia N. da Silva, Priscila S. e Souza, Marcelle M. Folena, Eduardo La M. da Silva

**Affiliations:** 1 Programa de Engenharia Ambiental, Escola Politécnica (PEA), Universidade Federal do Rio de Janeiro (UFRJ), Rio de Janeiro, RJ 21941-909, Brasil; 2 Instituto de Macromoléculas Professora Eloisa Mano (IMA), Universidade Federal do Rio de Janeiro (UFRJ), Rio de Janeiro, RJ 21941-598, Brasil; 3 Escola de Química, Universidade Federal do Rio de Janeiro (UFRJ), Rio de Janeiro, RJ 21941-909, Brasil

## Abstract

This study explores the addition of thyme and cinnamon essential oils into poly­(lactic acid) (PLA) and poly­(butylene adipate-*co*-terephthalate) (PBAT) blends to enhance their properties for sustainable packaging applications. The essential oils were added at 5 and 10 wt % into PLA/PBAT blends (80:20, wt %), and their effects on melt flow behavior, thermal and rheological properties, mechanical performance, and morphological structure were evaluated. Results indicate that the addition of essential oils substantially alters the properties of PLA/PBAT blends. Cinnamon oil showed a marked increase in the melt flow index, indicating a pronounced plasticizing effect, while thyme oil promoted enhanced ductility, increasing elongation at break from approximately 101% for the neat blend to about 171% at 10 wt % without compromising structural integrity. Thermal analyses revealed slightly enhanced thermal stability, with *T*_max_ shifting by approximately 5–8 °C, and modifications in dynamic crystallization behavior. Rheological assessments confirmed a reduction in complex viscosity, which decreased by roughly 40% at low frequencies, along with the predominance of viscous behavior in oil-containing samples. Mechanical tests showed that although essential oils generally reduce modulus and tensile strength—with Young’s modulus decreasing by about 15–25% depending on oil type—thyme oil contributes to higher elongation at break and improved toughness, increasing toughness by approximately 2.5-fold at 10 wt %. SEM analysis confirmed the immiscibility between PLA and PBAT and showed that essential oil incorporation alters the phase morphology. Overall, the findings highlight the potential of essential oils as functional additives capable of modulating the properties of biodegradable polymer blends for active packaging systems.

## Graphical abstract

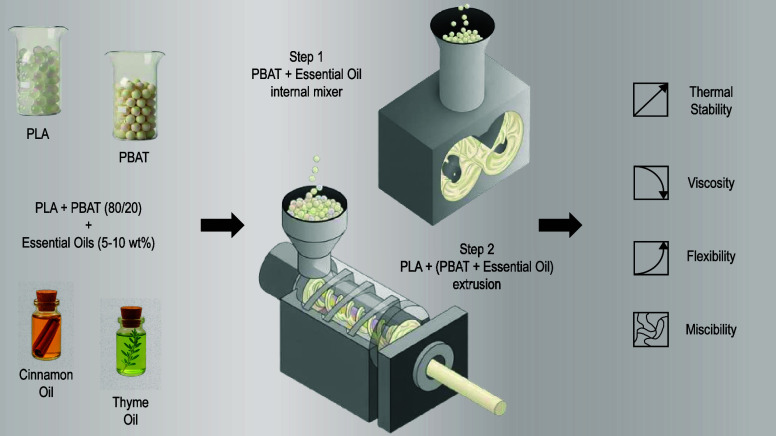

## Introduction

Environmental concern about plastic pollution has driven the development of biodegradable alternative plastic materials to replace conventional petroleum-derived polymers.^1,2^ Among the most prominent materials, poly­(lactic acid) (PLA) and poly­(butylene adipate-*co*-terephthalate) (PBAT) have emerged as promising alternatives due to their degradation capability under appropriate conditions and their potential combination to optimize mechanical and thermal properties.^3–6^ PLA, derived from renewable sources such as corn starch, exhibits high rigidity and mechanical strength, while PBAT provides flexibility and impact resistance. Typical PLA/PBAT blend ratios are optimized to balance rigidity and flexibility, often ranging from 80/20 to 50/50. This blend offers a viable approach for applications such as sustainable packaging.^7,8^

Beyond the increasing demand for the replacement of petrochemical-based materials, there is also a concern in the polymer industry regarding the properties of polymer blends to ensure improved performance.^9,10^ The addition of additives for the development of composites enhances the properties of biodegradable polymers, enabling these materials to compete with traditionally nonbiodegradable polymers materials established in the market.^11–13^ Among these innovations, active packaging has emerged as a promising application, incorporating functional compounds that interact with the packaged product to extend shelf life and maintain quality.^14,15^ Essential oils, for instance, have been widely explored as natural additives due to their antimicrobial and antioxidant properties, which can be effectively integrated into polymer matrices such as PLA and PBAT to improve their functional performance.^16–18^ By modifying the physicochemical properties of the polymer, these biodegradable active packaging systems not only reduce environmental impact but also offer a sustainable alternative to conventional packaging materials, aligning with the global trend toward circular economy strategies in the food and pharmaceutical industries.^19^

The incorporation of essential oils into polymeric matrices has been widely studied to confer additional functionalities, such as antimicrobial, antioxidant, and plasticizing properties.^20,21^ In this context, thyme (*Thymus vulgaris*) and cinnamon (*Cinnamomum cassia*) essential oils stand out due to their well-known antimicrobial activities and potential for extending the shelf life of packaged foods.^21,22^ Additionally, these oils act as natural plasticizers, reducing viscosity and facilitating the processing of PLA/PBAT composites, as evidenced by the increased melt flow index (a measure of the ease of flow of a thermoplastic’s melt) of blends containing these additives.^23,24^

Despite the advantages associated with the use of essential oils, technical challenges still need to be overcome, such as the compatibility between polymers and oils and the thermal stability of these compounds during processing.^25,26^ Studies indicate that the addition of compatibilizing agents, such as maleic anhydride, can improve oil dispersion in the polymer matrix, promoting better miscibility between the PLA and PBAT phases.^27^ Moreover, the thermal stability of essential oils may be a limiting factor for their use in processes involving intense heating and shear forces, requiring the development of strategies to minimize the degradation of these compounds during the processing stage.^28,29^

To develop sustainable and efficient biodegradable packaging, this study evaluates the effects of the addition of thyme and cinnamon essential oils into PLA/PBAT composites. Specifically, it examines how these oils affect thermal properties, rheological behavior, and mechanical properties. Although several studies have incorporated essential oils into PLA or PBAT using solution casting or fiber production, only a few have addressed the direct incorporation of essential oils into PLA/PBAT blends via twin-screw extrusion, combined with a two-roll milling step prior to final extrusion, along with an integrated evaluation of oil retention during melt processing and its effects on the mechanical, barrier, and rheological properties of the resulting materials. Therefore, the research also assesses the composites’ potential for packaging applications, exploring the synergistic benefits of this novel essential oil combination.

## Materials and Methods

### Materials

This research aims to evaluate the addition of two distinct essential oils, thyme and cinnamon, into polymeric blends of PLA and PBAT, focusing on their effects on polymer compatibility and properties.

The polymers were PLA (grade FC50010) supplied by Earth Renewable Technologies (ERT) and PBAT (grade F Blend C1200) provided by Badische Anilin- and Sodafabrik (BASF).

The essential oils employed were white thyme essential oil (*T. vulgaris* flower/leaf oil), extracted from leaves through steam-distillation, and cinnamon bark essential oil (*C. cassia* oil), obtained from bark and twigs by steam distillation. Both essential oils were supplied by Quinarí and were provided with manufacturer-certified purity specifications.

### Methodology

Figure 1 illustrates a summary of the methodological steps employed in this study for the incorporation of essential oils into the polymeric blends.

**Figure 1:**
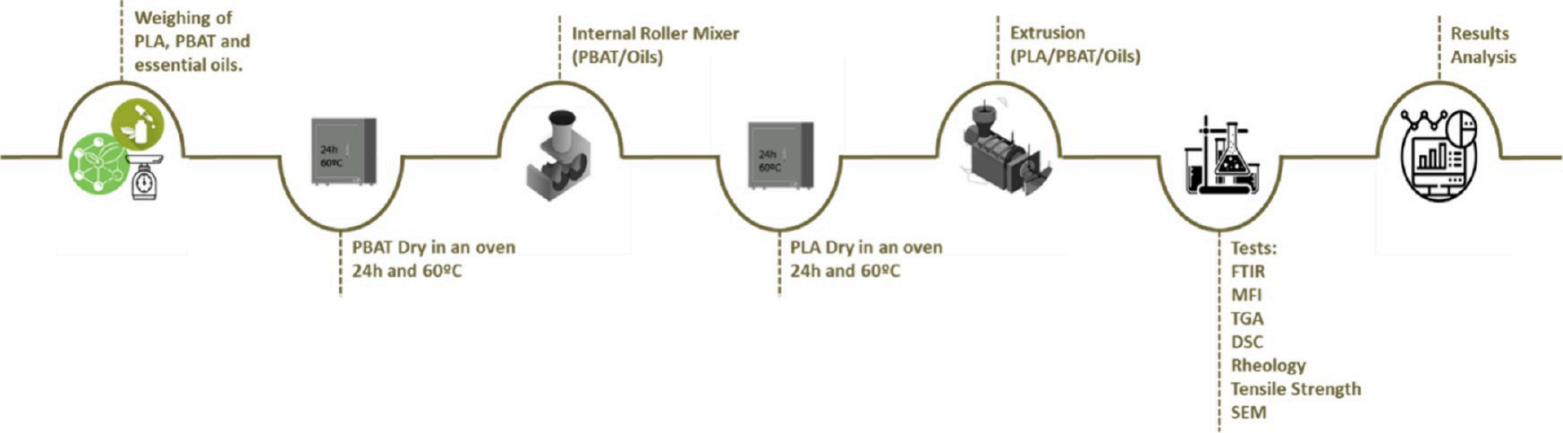
Methodology applied to the evaluation of biopolymer composites with essential oils.

The processing of biopolymer blends with essential oils was designed considering the properties of both the essential oils and the biopolymers. The first stage involved blending the essential oil with PBAT, which has a lower melting temperature, enabling processing under milder thermal conditions. Subsequently, the PBAT/oil composite was combined with PLA to produce the targeted PLA/PBAT/oil composite. The incorporation of the oils into the polymer matrices was performed in two distinct stages to preserve their properties, given their limited thermal stability.

The mixing of PBAT with the essential oils was carried out using a Thermo Scientific HAAKE Rheomex internal mixer, model PTW16 (Thermo Fisher Scientific, Karlsruhe, Germany), equipped with a roller screw. The processing parameters included a temperature of 90 °C, a rotation speed of 60 rpm, and a mixing duration of 10 min. Before processing, PBAT was dried in an oven at 60 °C for 24 h. After the incorporation of the oil, a second processing step was conducted using a Thermo Scientific twin-screw extruder, model HAAKE Rheomex OS PTW16, equipped with a 16 mm screw diameter, a 40:1 L/D ratio, and a maximum rotation speed of 250 rpm. For this step, the extruder was operated at a screw speed of 300 rpm, a feeder rotation speed of 32 rpm, and a temperature profile ranging from 90 to 150 °C (90/130/140/145/135/135/135/135/140/150 °C). Prior to extrusion, PLA was dried under the same conditions as PBAT in the first step (60 °C for 24 h), whereas the PBAT/oil composite was not dried to prevent the volatilization of essential oil compounds.

The experimental design included two process variables: oil content (5 and 10 wt %) and type of oil (thyme and cinnamon). Additionally, two control experiments were conducted: one with a PLA/PBAT blend without oil and another with neat PLA. In all blend formulations, the PLA/PBAT mass ratio was maintained at 80:20 wt %. Table 1 summarizes the experimental design and the mass percentages for each composition.

**Table 1:** Experimental Design for the Development of PLA/PBAT/Oil Blends^a^

experiment	PLA (wt %)	PBAT (wt %)	oil (wt %)
PLA	100	0	0
PLA/PBAT	80	20	0
PLA/PBAT/T5	76	19	5
PLA/PBAT/C5	76	19	5
PLA/PBAT/T10	72	18	10
PLA/PBAT/C10	72	18	10

a *T* = thyme oil and C = cinnamon oil.

### Material Characterization

#### Fourier Transform Infrared Spectroscopy (FTIR)

Fourier transform infrared spectroscopy (FTIR) was performed using a PerkinElmer *Frontier FTIR/FIR* spectrometer (PerkinElmer Inc., Waltham, Massachusetts, USA) equipped with an attenuated total reflectance (ATR) accessory. Spectra were collected in the range of 4000–400 cm^–1^ with a spectral resolution of 4 cm^–^^1^ and 60 scans for each measurement. The ATR crystal was cleaned with isopropanol before each acquisition, and background spectra were recorded prior to sample analysis.

#### Melt Flow Index (MFI)

The MFI analyses were performed on a Dynisco Instruments LMI 4000 (Dynisco Instruments, Franklin, Massachusetts, USA) melt flow index meter, following the ASTM 1238 standard. Before the test, the analyzed pellets were dried in an oven at 60 °C for 24 h, and the parameters used in the MFI equipment during the test were as follows: temperature of 180 °C, load of 2.16 kg, melting time of 240 s, and cutting time of 15 s. Tests were performed using the extruded pellets. Due to specific behaviors in some composites, eight cuts were taken every 15 s, except in samples with high MFI values, where four cuts were made.

#### Thermogravimetric Analysis (TGA)

Thermogravimetric analyses were conducted using a TA Instruments Q500 thermogravimetric analyzer (TA Instruments, New Castle, Delaware, USA), according to the ASTM E1131 standard. Pellets and an inert nitrogen (N_2_) atmosphere were used for the tests, with the following test conditions: temperature range analyzed 25 to 700 °C and heating rate of 10 °C/min. This technique was applied to evaluate the behavior of commercial polymers and blends processed by extrusion, with six repetitions being performed for each sample. The temperatures at weight loss onset (*T*_onset_) and maximum degradation rate (*T*_max_) were determined by the first derivative.

#### Differential Scanning Calorimetry (DSC)

Differential scanning calorimetry was measured using a TA Instruments Q1000 calorimeter (TA Instruments, New Castle, Delaware, USA). These tests were performed with a commercial polymer and blends produced. A first heating from −50 to 250 °C was performed at a rate of 10 °C/min, with an isotherm of 1 min; then, the material was subjected to rapid cooling to −50 °C; subsequently, there was a second heating from −50 to 250 °C at a rate of 10 °C/min; a second cooling from 250 to −50 °C was performed at a rate of 10 °C/min; and finally, a third heating from −50 to 250 °C at a rate of 10 °C/min was performed again.

Thermal responses from DSC curves were used to calculate the crystallinity degrees, obtained from third heating of pure PLA and PLA in processed composites using eq 1:

$$\chi_{c}=\frac{{\Delta H}_{m}-{\Delta H}_{c}}{\left({{{\Delta H}_{m}}^{\infty }}_{PLA}\right)}$$ 1

In this equation, Δ*H*_m_ is the enthalpy variation during melting, Δ*H*_c_ is the enthalpy variation during crystallization, $\Delta {{H_{m}}^{\infty }}_{\mathrm{PLA}}$ represents the theoretical enthalpy for 100% crystalline PLA (93 J/g), and %PLA is the mass fraction of PLA in the sample.^30^

#### Oscillatory Rheology

Oscillatory rheology was performed on a TA Instrument AR2000 oscillatory rheometer at 190 °C and with 25 mm-diameter parallel plate geometry. The linear viscoelastic zone was assessed by performing strain sweep tests from 0.1 to 100% at 1 Hz. Frequency sweep tests from 0.1 to 600 rad s^–1^ were performed at 1% strain under N_2_ atmosphere.

The power law model (eq 2) was used to evaluate the flow behavior of the materials.

$$\eta \left(\mathrm{\gamma ̇}\right)=K^{*}{\mathrm{\gamma ̇}}^{n-1}$$ 2

where η is the complex viscosity, γ̇ is the shear rate, *K* is the consistency index, and *n* is the power law index.

#### Tensile Strength

The tensile strength tests were performed on a Universal Testing Machine EMIC DL series (EMIC Equipamentos e Sistemas de Ensaio, São José dos Pinhais, PR, Brazil). Type V test specimens were tested, according to the ASTM D638 standard, at a speed of 1 mm/min (defined after preliminary tests).

An Xplore IM12 injection molding machine was used for the test. Before molding the test specimens, the processed pellets were dried in an oven at 60 °C for 24 h and mixed using an Xplore MC 15 HT conical twin-screw extruder, with a temperature of 150 °C in the three heating zones, for 1 min. The injection was at 170 °C with a mold temperature of 40 °C, filling pressure of 6 bar for 5 s, holding pressure of 5 bar for 3 s, and holding pressure of 5 bar for 2 s.

#### Scanning Electron Microscopy (SEM)

The fractured surface morphology of the materials was investigated using a TESCAN MIRA fourth-generation LMU field-emission scanning electron microscope (TESCAN ORSAY HOLDING, Brno, Czech Republic). The specimens were frozen in liquid nitrogen and fractured manually. Samples were sputter-coated with a thin gold layer prior to imaging.

All measurements were conducted in replicates to ensure accuracy and consistency of the results. However, the experimental design of this study focused on comparative analysis of processing effects and material performance rather than on a statistical treatment of the properties. Accordingly, the data were collected and recorded in the format provided by each instrument, which includes averaged values and standard deviations but not raw replicate-level data sets suitable for post hoc statistical modeling. Although formal hypothesis testing was not incorporated into the original methodological scope, the differences observed between the formulations are substantially greater than typical instrumental variability, supporting the robustness of the conclusions. This point has now been clarified in the manuscript.

## Results and Discussion

### Fourier Transform Infrared Spectroscopy (FTIR)

Figure 2 presents the FTIR spectra of neat PLA, the PLA/PBAT (80/20) blend, and the systems containing thyme (T5, T10) and cinnamon (C5, C10) essential oils. The characteristic absorption band of the ester carbonyl group of PLA, located at 1750–1745 cm^–1^, was observed in all samples; however, the incorporation of essential oils produced a slight shift of this band toward lower wavenumbers (up to −6 cm^–1^ for T10), accompanied by an increase in bandwidth. These two effects suggest specific interactions between the carbonyl groups of PLA and the oxygenated functional groups present in the essential oils, particularly phenolic hydroxyls in thyme oil and conjugated carbonyls in cinnamon oil.

**Figure 2:**
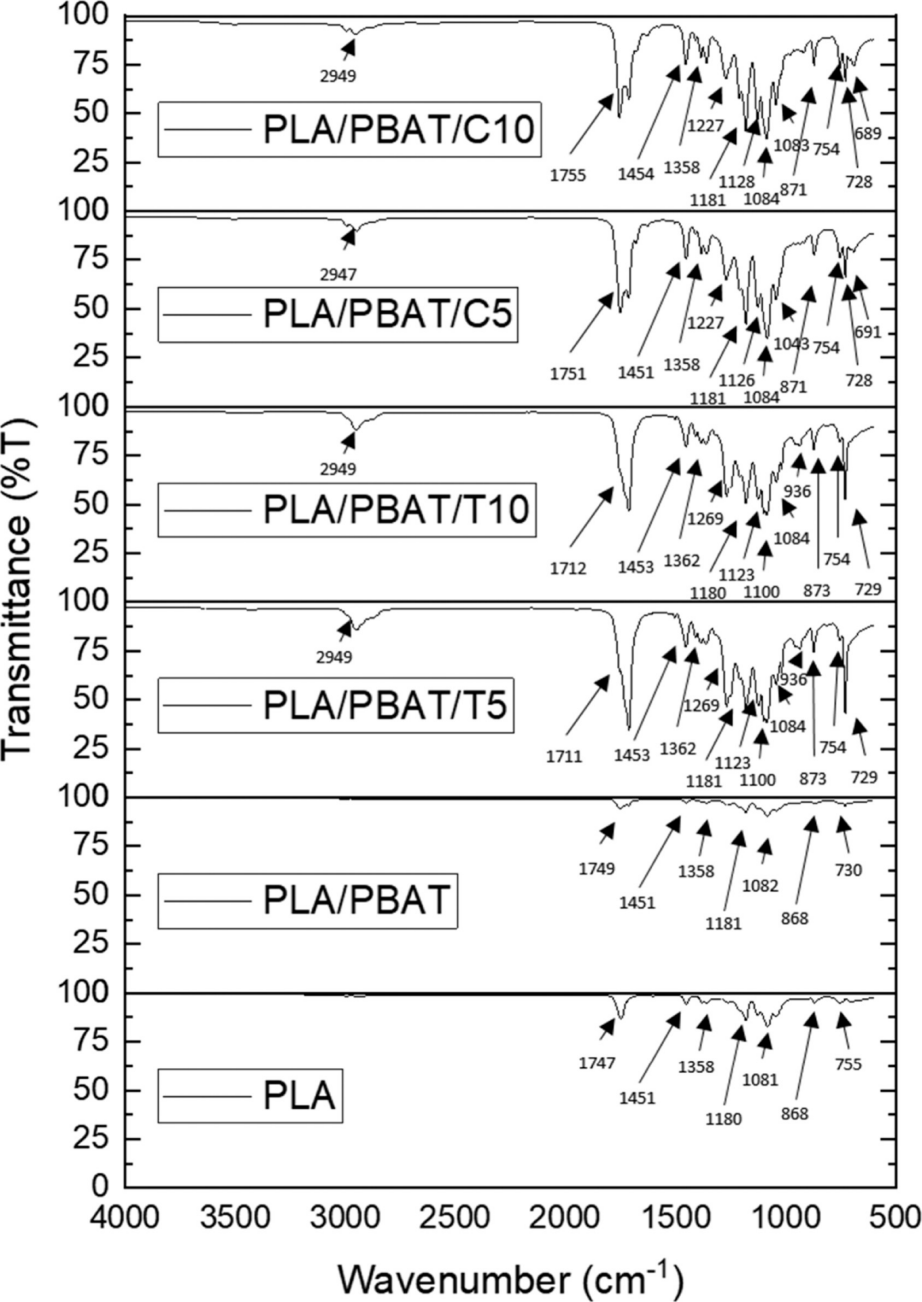
FTIR spectra of PLA, PLA/PBAT, and oil-containing formulations.

The PLA/PBAT blend exhibited additional signals at 1600–1500 cm^–1^ and at approximately 720 cm^–1^, which correspond to the aromatic ring of the terephthalate segments of PBAT and were used to confirm blend composition. Samples containing thyme oil (T5 and T10) showed a moderate increase in the intensity of the aromatic region (1620–1500 cm^–1^) and in the C–O stretching region (1250–1000 cm^–1^), consistent with the presence of phenolic constituents (thymol/carvacrol). In contrast, cinnamon-containing films displayed a distinct shoulder near 1680–1670 cm^–1^, attributable to the conjugated C=O of cinnamaldehyde, confirming the effective incorporation of this compound into the polymer matrix.

A weak and broad band between 3500 and 3200 cm^–1^ appeared in the T10 and C10 formulations, which may indicate either the presence of free phenolic O–H or enhanced water adsorption promoted by the oils. Overall, the FTIR results confirm the chemical incorporation of the essential oils and suggest the formation of weak intermolecular interactions with the PLA-rich phase of the blend, which is consistent with the changes observed later in the thermal, mechanical, and rheological properties.

These FTIR results are consistent with observations reported in similar PLA-based systems containing essential oils. Correa-Pacheco et al. found that the incorporation of cinnamon oil into PLA/PBAT produced carbonyl band shifts toward lower wavenumbers and a characteristic shoulder near 1680 cm^–1^, matching the behavior observed in our C5 and C10 films. Likewise, studies on PLA composites containing thymol have reported increases in the aromatic and C–O regions and the appearance of a broad O–H band due to hydrogen-bond interactions with the polyester matrix. Together, these findings confirm that the spectral changes detected in our thyme- and cinnamon-containing formulations fall within the expected and well-documented FTIR responses of PLA systems modified with oxygenated essential oils. Intermolecular interactions occur between oxygenated compounds and the PLA-rich phase.^20,31^

### Melt Flow Index (MFI)

After processing, the flow properties of PLA/PBAT with oil-based blends were determined by MFI analysis, and the results are shown in Table 2.

**Table 2:** MFI Results of PLA and PLA/PBAT/Oil Composites

experiment	MFI (g·10^–1^)
PLA	10.33 ± 0.50
PLA/PBAT	11.76 ± 0.48
PLA/PBAT/T5	19.57 ± 1.03
PLA/PBAT/T10	20.12 ± 0.80
PLA/PBAT/C5	37.56 ± 0.96
PLA/PBAT/C10	120.27 ± 7.96

Initially, it is noteworthy that the flow behavior of PLA remained stable after processing in the extruder when compared to the commercial PLA tested under the same conditions. Considering the standard deviations, the neat PLA has an MFI value of 9.84 ± 0.22, whereas after processing, its MFI was 10.33 ± 0.50. This result shows that the processing conditions did not lead to significant PLA degradation.

When PBAT was added into the PLA matrix at a mass ratio of PLA/PBAT 80/20 (wt %), the impact on the flow property was minimal. Despite PBAT having a melt flow index approximately twice that of PLA under the tested conditions, the results for the PLA/PBAT blend revealed that its behavior remained predominantly similar to that of neat PLA. Therefore, at the investigated compositions, PBAT did not significantly alter the flow behavior of the PLA matrix, as demonstrated also by previous research.^32^

Conversely, the addition of natural oils to the composites significantly increased their melt flow properties, both with thyme oil and cinnamon oil. For the thyme oil-containing samples, the MFI increased by 66% compared to the PLA/PBAT blend without oil. However, no significant change was observed with increasing thyme oil content. In contrast, cinnamon oil not only caused a more pronounced variation in MFI values but also induced a severe change in the material’s behavior at higher oil contents. These findings suggest that, under the test conditions, both oils act as plasticizers by reducing the polymer’s viscosity. Additionally, cinnamon oil exhibited a greater influence on the flow properties of the composites compared to thyme oil.

The addition of essential oils into polymeric composites generally reduces the viscosity of the polymer matrix, leading to an increase in MFI values. This effect results from the interaction between the polymer matrix and the added oil, as reported in the literature. Some authors suggest that the higher the dispersion and interaction of the oil with the polymer chains, the greater its plasticizing effect, which consequently reduces intermolecular forces between the polymer chains and enhances their mobility under processing conditions.^20,33^ Therefore, the addition of thyme and cinnamon essential oils significantly increases the melt flow index of PLA/PBAT blends, acting as plasticizers.

### Thermogravimetric Analysis (TGA)

The TGA curves for the produced systems are presented in Figure 3.

**Figure 3:**
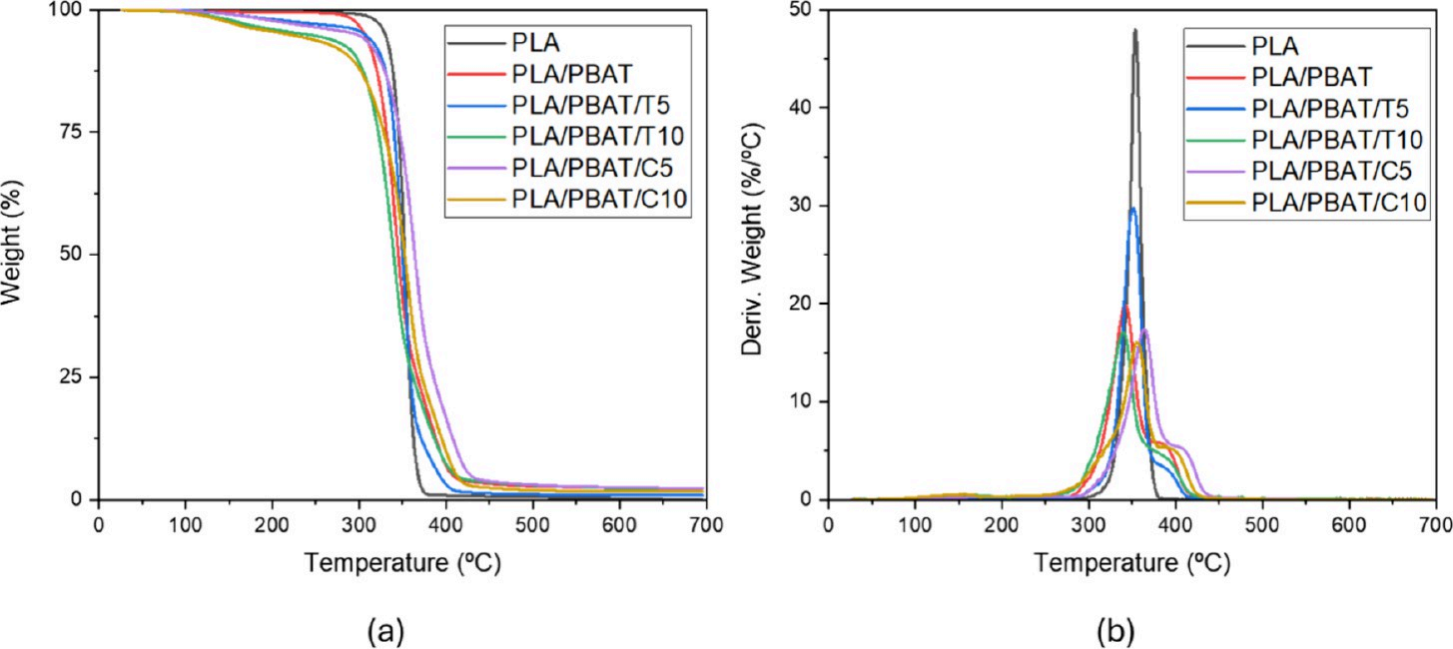
TGA (a) mass loss and (b) derivative mass loss curves of the processed blends.

From the curves, it is evident that the thermal stability of the PLA matrix does not vary significantly in the presence of the PBAT phase or when essential oils are incorporated into the PLA/PBAT blends. To better understand the thermal degradation properties, the temperatures corresponding to the maximum degradation rate (*T*_max_) were determined (Table 3).

**Table 3:** Maximum Degradation Temperature Rate (*T*_max_) of Neat PLA and PLA/PBAT Compositions from TGA and DTG Curves

	*T*_max_ (°C)		
experiment	peak 1oil	peak 2PLA	peak 3PBAT
T^a^	104 ± 1		
C	144 ± 1		
PLA		352 ± 2	
PLA/PBAT		343 ± 1	374 ± 3
PLA/PBAT/T5	174 ± 7	354 ± 7	379 ± 3
PLA/PBAT/T10	171 ± 6	348 ± 9	377 ± 6
PLA/PBAT/C5	172 ± 2	361 ± 9	389 ± 4
PLA/PBAT/C10	171 ± 8	355 ± 3	381 ± 4

a T = thyme oil and C = cinnamon oil.

Despite certain limitations, the analysis of the thermogravimetric curves allowed the identification of three distinct mass loss phases, related to essential oil, PLA, and PBAT. The occurrence of multiple degradation peaks suggests a sequential or stepwise degradation process, a characteristic behavior of multiphase systems. It is noteworthy that the essential oils exhibited their maximum thermal degradation peaks at higher temperatures compared to the raw materials. This increase in degradation temperature rate after oil addition into the blends may indicate an effective interaction between the essential oils and the polymeric matrices, leading to an improvement in the thermal properties of the materials. This thermal stabilization effect may arise from hydrogen bonding, dipole–dipole, and hydrophobic interactions between the oils and the polymer chains, which enhance cohesion and restrict molecular mobility, thereby delaying degradation.

As previously mentioned, regarding the interaction between the two primary polymers, the addition of PBAT to PLA resulted in a reduction in PLA’s maximum degradation temperature rate. This phenomenon can be attributed to the physical and chemical interactions between PLA and PBAT chains, where the presence of PBAT may enhance molecular mobility and consequently facilitate the degradation of the PLA phase. Recent studies on the miscibility and degradation dynamics of PLA/PBAT-based blends corroborate this result, suggesting that the interaction between the polymeric phases is relatively weak, which may promote phase segregation and, consequently, a reduction in PLA’s thermal stability when combined with PBAT.^23^

Finally, the samples containing essential oils exhibited variations in degradation temperatures compared to PLA/PBAT composition, highlighting the effectiveness of these additives as moderate stabilization effect. The essential oils appear to interact with the polymer chains, forming a protective barrier that decreases the thermal degradation rate. This enhanced thermal stabilization effect allows the system to maintain its properties at higher temperatures, a crucial aspect for applications where thermal resistance is a fundamental requirement.

Among the evaluated systems, the PLA/PBAT/C5 blend exhibited the most pronounced stabilization effect, with the degradation peaks shifted to higher temperatures compared to the other compositions. This result suggests that a moderate concentration of cinnamon oil is sufficient to promote effective interactions with the polymer chains, enhancing cohesion without significantly disturbing the structural organization of the matrix.

### Differential Scanning Calorimetry (DSC)

The DSC results were evaluated from the heating and cooling curves and the data were arranged in Tables 4, 5, 6, and 7.

**Table 4:** Thermal Properties Determined from DSC Curves for the Second Heating^a^

	second heating						
experiment	*T*_g_ (°C)	*T*_c1_ (°C)	Δ*H*_c1_ (J/g)	*T*_c2_ (°C)	Δ*H*_c2_ (J/g)	*T*_m_ (°C)	Δ*H*_m_ (J/g)
neat PLA	62.1 ± 0.1	97.0 ± 0.1	55.0 ± 0.1	156.8 ± 0.2	8.5 ± 0.3	173.3 ± 0.4	64.9 ± 0.7
PLA/PBAT	61.2 ± 0.2	93.5 ± 0.4	38.4 ± 0.6	155.9 ± 0.2	7.5 ± 0.3	172.6 ± 0.2	62.1 ± 0.3
PLA/PBAT/T5	58.7 ± 0.9	93.0 ± 0.1	39.5 ± 1.4	155.0 ± 0.1	7.4 ± 0.3	172.3 ± 0.1	54.9 ± 0.3
PLA/PBAT/T10	55.6 ± 0.5	91.5 ± 0.2	31.0 ± 1.8	153.7 ± 0.4	6.1 ± 0.3	171.1 ± 0.1	44.3 ± 1.7
PLA/PBAT/C5	57.9 ± 0.5	91.4 ± 0.7	33.3 ± 0.9	153.9 ± 0.1	6.4 ± 0.5	171.3 ± 0.4	49.3 ± 2.0
PLA/PBAT/C10	52.8 ± 0.1	87.9 ± 0.1	36.4 ± 0.9	150.4 ± 0.2	5.8 ± 0.3	169.0 ± 0.1	53.0 ± 0.2

a *T*_g_ = glass transition temperature; *T*_c_ = crystallization temperature; Δ*H*_c_ = crystallization enthalpy; *T*_m_ = melting temperature; and Δ*H*_m_ = melting enthalpy.

**Table 5:** Thermal Properties Determined from the DSC Curves for Controlled Cooling^a^

	cooling	
experiment	*T*_c_ (°C)	Δ*H*_c_ (J/g)
PLA		
PLA/PBAT	109.0 ± 0.2	39.4 ± 1.4
PLA/PBAT/T5	103.1 ± 0.1	36.3 ± 0.6
PLA/PBAT/T10	96.4 ± 0.7	15.2 ± 0.4
PLA/PBAT/C5	104.7 ± 0.1	36.7 ± 1.5
PLA/PBAT/C10	94.4 ± 0.2	8.9 ± 0.5

a *T*_c_ = crystallization temperature; and Δ*H*_c_ = crystallization enthalpy.

**Table 6:** Thermal Properties Obtained from the DSC Curves for the Third Heating Cycle^a^

	third heating						
experiment	*T*_g_ (°C)	*T*_c1_ (°C)	Δ*H*_c1_ (J/g)	*T*_c2_ (°C)	Δ*H*_c2_ (J/g)	*T*_m_ (°C)	Δ*H*_f_ (J/g)
PLA	62.0 ± 0.2	97.5 ± 0.1	53.3 ± 0.3	157.0 ± 0.2	8.6 ± 0.4	173.4 ± 0.4	64.8 ± 0.8
PLA/PBAT	62.1 ± 0.3					174.2 ± 0.3	48.5 ± 0.2
PLA/PBAT/T5	60.2 ± 0.1	94.3 ± 0.1	5.4 ± 0.2	158.7 ± 0.1	2.6 ± 0.2	173.0 ± 0.1	52.7 ± 0.4
PLA/PBAT/T10	56.9 ± 1.7	92.3 ± 0.6	14.6 ± 0.9	155.4 ± 0.7	5.0 ± 0.2	171.9 ± 0.3	45.0 ± 1.5
PLA/PBAT/C5	61.5 ± 0.2			159.7 ± 0.3	0.9 ± 0.3	172.9 ± 0.4	44.9 ± 2.5
PLA/PBAT/C10	55.7 ± 0.5	90.0 ± 0.1	27.9 ± 0.1	152.8 ± 0.1	6.2 ± 0.1	170.3 ± 0.1	53.0 ± 0.1

a *T*_g_ = glass transition temperature; *T*_c_ = crystallization temperature; Δ*H*_c_ = crystallization enthalpy; *T*_m_ = melting temperature; and Δ*H*_m_ = melting enthalpy.

Table 4 presents the data from second heating, conducted after rapid cooling (quenching). In those curves, two exothermic peaks were observed, corresponding to crystallization, along with an endothermic peak associated with the melting of the material. This curve, after abrupt cooling, allows a detailed analysis of the glass transition temperature (*T*_g_) of the studied polymers. It was observed that the addition of essential oils significantly reduced the *T*_g_ of PLA, indicating increased polymer chain mobility and resulting in a material with lower rigidity at lower temperatures. Among the evaluated essential oils, cinnamon oil exhibited the most pronounced effect in reducing the PLA matrix *T*_g_ compared to thyme oil.

After the second heating, the samples were cooled in a controlled manner to evaluate the behavior of the melt during cooling, as shown in Table 5.

From the results shown in the table, it is highlighted that neat PLA did not exhibit an exothermic crystallization peak during cooling, indicating its predominantly amorphous nature under the applied conditions, where it is similar to other studies' results, and it can be explained as a result of PLA’s stereochemical structure and poor chain mobility that changes with nucleating agents, plasticizers, or blends with other polymers.^34^ However, the addition of PBAT into the matrix led to the formation of a crystallization peak in all composites, demonstrating a higher tendency for crystallization induced by the presence of the copolymer. As the essential oil content increased, the enthalpy associated with this crystallization peak decreased significantly. In systems containing 10 wt % oils, this reduction was particularly pronounced, resulting in an approximately 4-fold decrease in crystallization enthalpy compared to other compositions. This behavior may be related to the plasticizing effect of the oils, which interferes with the polymer chain rearrangement during cooling, hindering the formation of ordered crystalline structures.

The third heating curve (Table 6) was obtained after controlled cooling and enabled a more detailed analysis of the thermal behavior of the materials.

In these curves, the PLA/PBAT systems did not show exothermic crystallization peaks during heating, suggesting that crystalline rearrangement predominantly occurred during the cooling stage. With increasing essential oil content in the blends, the higher free volume induced by the plasticizing effect hindered chain packing, thus reducing the crystallization tendency of the system rather than promoting it.

For pure PLA, which did not crystallize during cooling, two crystallization peaks were observed during heating, with higher enthalpy variations than the other analyzed systems. This behavior can be attributed to the greater rigidity of pure PLA, which prevents crystallization during cooling but allows for more significant polymer chain rearrangement when subjected to a controlled heating rate.

Table 7 shows the crystallinity degree from DSC data for the third heating cycle.

**Table 7:** Degree of Crystallinity from the Third Heating Cycle

experiment	crystallinity (%)
PLA	1.6 ± 0.6
PLA/PBAT	13.2 ± 1.2
PLA/PBAT/T5	13.5 ± 1.2
PLA/PBAT/T10	16.1 ± 0.6
PLA/PBAT/C5	11.5 ± 1.1
PLA/PBAT/C10	10.7 ± 0.2

It was observed that the addition of PBAT into the PLA matrix resulted in a significant increase in polymer crystallinity under the processing conditions applied in this study. This behavior suggests a possible interaction between the polymer phases that favored the rearrangement of PLA chains.

The incorporation of essential oils also affected the crystalline behavior of the PLA/PBAT bend, with the magnitude and direction of the effect depending on the type of oil. In blends containing 10 wt % thyme oil, a slight increase in crystallinity was observed, whereas the addition of 10 wt % cinnamon oil led to a marked reduction. The reduction induced by cinnamon oil is consistent with its plasticizing effect, in which enhanced chain mobility restricts efficient crystalline packing. Conversely, the increase promoted by thyme oil appears to be an exception and may be related to modifications in the cold crystallization process. Previous studies reported crystallinity values of 31% for neat PLA, 35% for PLA with carvacrol, and 33% for PLA with thymol, indicating that these additives may slightly affect the crystalline fraction of PLA, although the authors do not consider this a significant modification on PLA crystallinity behavior;^34^ beside that, other research demonstrated that thyme addition to PLA modifies its cold crystallization temperature, suggesting that such molecules influence chain ordering during crystallization.^35^ This effect could be attributed to the chemical structure of thymol, in which the hydroxyl group and the aromatic ring allow intermolecular interactions with PLA ester groups, altering chain mobility and promoting changes in nucleation and crystallization pathways.

### Oscillatory Rheology

The oscillatory rheology test was conducted on the granules obtained after extrusion. Figure 4 presents the flow behavior of the processed systems, from complex viscosity curves of the processed samples highlighting significant differences in the rheological behavior of the systems.

**Figure 4:**
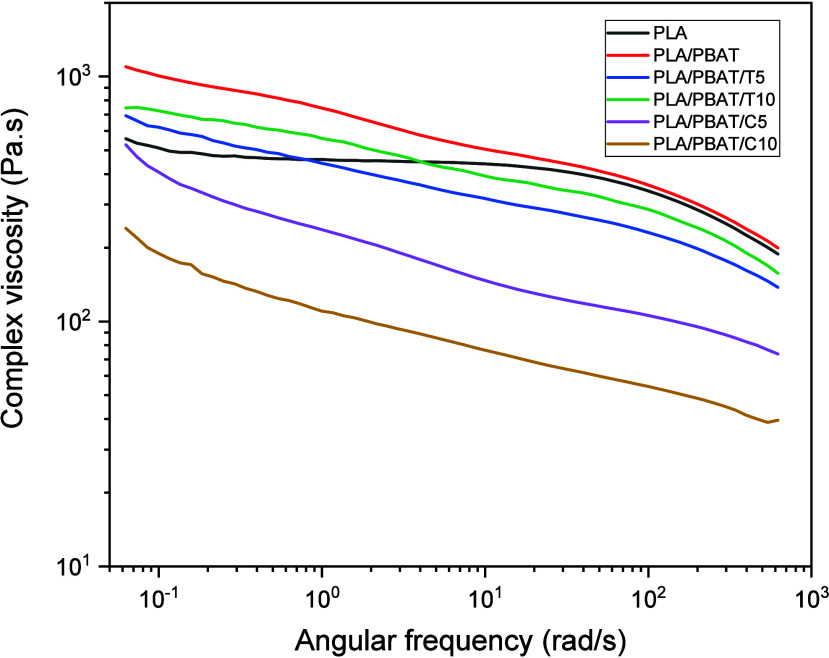
Complex viscosity curves of the processed composites.

In the low-frequency region, the addition of PBAT into the PLA matrix results in an increase in material viscosity. This behavior suggests a reduction in the mobility of PLA polymer chains, as the introduction of PBAT promotes stronger intermolecular interactions, restricting the flow of PLA chains.

It is important to note that PLA exhibits Newtonian behavior up to approximately 10 rad/s. The addition of PBAT induces pseudoplastic behavior.

The addition of essential oils, which act as plasticizing agents, modifies this scenario. These oils enhance the viscous behavior of the systems by promoting polymer chain mobility, facilitating movement, and consequently improving flow under stress. As a result, the systems processed with cinnamon oil exhibit more pronounced viscous behavior, demonstrating the efficiency of this oil in reducing flow resistance.

In the high-frequency region, the analysis of the viscosity curves reveals distinct behavior. PLA/PBAT composites exhibit a sharp decrease in viscosity, more pronounced than that of neat PLA, indicating that under higher stress conditions, PBAT chains align more efficiently with PLA chains. This alignment facilitates the joint mobility of polymer chains, reducing viscosity at high frequencies. A similar trend is observed in composites containing essential oils, which maintain lower viscosity values even at high frequencies due to the continued plasticizing effect of the oils, which enhances chain mobility.^36^ However, while the PLA/PBAT system at high frequencies tends to behave similarly to PLA, samples containing essential oils show an even more pronounced viscosity reduction, suggesting that the plasticizing effect prevails under these conditions.

From the complex viscosity curves (Figure 4), the consistency index (*k*) and the power law index (*n*) were determined using the power law equation (eq 2), as presented in Table 8.

**Table 8:** Consistency Index (*k*) and Power Law Index (η) Obtained from the Rheology Results for the Processed Samples

experiment	consistency index (*K*)	power law index (*n*)
PLA	463	0.92
PLA/PBAT	726	0.83
PLA/PBAT/T5	444	0.85
PLA/PBAT/T10	544	0.85
PLA/PBAT/C5	243	0.81
PLA/PBAT/C10	117	0.82

The consistency index values corroborate the previously discussed results, evidencing an increase in PLA viscosity with the addition of PBAT. Additionally, the plasticizing effect induced by essential oils leads to a viscosity reduction in the systems, with this effect being more pronounced in formulations containing cinnamon oil.

Regarding the power law index, all samples fall within the characteristic range of pseudoplastic materials. The incorporation of PBAT into the PLA matrix enhances this behavior, shifting the systems further from a Newtonian response and accentuating the pseudoplastic nature. This implies greater sensitivity of the samples to shear rate.

As demonstrated from *G*′ and *G*″ curves versus frequency (Figure 5), no intersection points between the storage modulus (*G*′) and loss modulus (*G*″) was identified across the evaluated angular frequency range, indicating that the viscoelastic behavior of the samples does not exhibit a clear transition between an elastic-dominant and a viscous-dominant regime. Furthermore, in all samples, the loss modulus remained higher than the storage modulus, demonstrating that viscous behavior prevails over elastic behavior throughout the investigated frequency range. This behavior suggests that under these dynamic conditions, the samples predominantly deform irreversibly, absorbing more mechanical energy as dissipation (viscous behavior) rather than storing it elastically.

**Figure 5:**
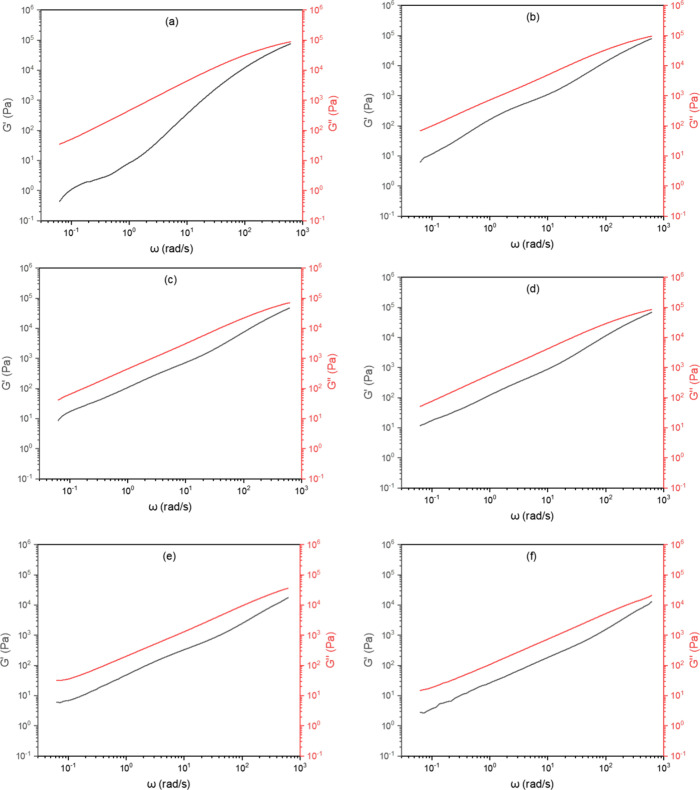
Storage modulus and loss modulus curves obtained from oscillatory rheology curves for (a) PLA, (b) PLA/PBAT, (c) PLA/PBAT/T5, (d) PLA/PBAT/T10, (e) PLA/PBAT/C5, and (f) PLA/PBAT/C10.

These results reinforce the hypothesis that the presence of PBAT and the addition of essential oils contribute to reducing the structural stiffness of the systems, enhancing plasticity and facilitating deformation under cyclic loading. The absence of a crossover points between *G*′ and *G*″ indicates that the material does not reach an elasticity-dominated regime at any of the evaluated frequencies, further reinforcing the plasticizing effect of the additives.

It is important to note that for plastic packaging production, a balance between elastic and viscous properties is desirable. Therefore, based on the obtained rheological results, it is expected that the systems containing thyme oil will demonstrate better performance in film production.

### Tensile Strength

The mechanical properties of the composites were determined from stress–strain curves. The results are presented in Tables 9 and 10.

**Table 9:** Mechanical Properties Obtained from the Tensile Strength Test Curves

experiment	elastic modulus (MPa)	yield strength (MPa)	yield elongation (%)
PLA	2226 ± 126	73 ± 2	7.3 ± 0.9
PLA/PBAT	1783 ± 56	56 ± 1	6.2 ± 0.3
PLA/PBAT/T5	1653 ± 140	44 ± 4	5.6 ± 0.3
PLA/PBAT/T10	1681 ± 114	41 ± 3	5.3 ± 0.1
PLA/PBAT/C5	1684 ± 178	46 ± 3	5.4 ± 0.2
PLA/PBAT/C10	1544 ± 98	36 ± 2	5.1 ± 0.1

**Table 10:** Mechanical Properties from Tensile Strength Curves

experiment	strain at break (%)	stress at break (MPa)	toughness (MPa)
PLA	9 ± 1	60 ± 4	
PLA/PBAT	101 ± 32	14 ± 3	1453 ± 13
PLA/PBAT/T5	159 ± 26	11 ± 3	1310 ± 134
PLA/PBAT/T10	171 ± 11	11 ± 4	1186 ± 60
PLA/PBAT/C5	82 ± 37	11 ± 2	1086 ± 117
PLA/PBAT/C10	100 ± 51	8 ± 2	936 ± 26

Table 9 presents the mechanical properties obtained at the yield tensile stress point for the produced systems. The elastic modulus, measured in the elastic deformation region, reflects the initial resistance of the materials to tensile loading while maintaining elastic behavior. As expected, and consistent with previous analyses, the results indicate that neat PLA exhibits high tensile strength at low deformations due to its higher elastic modulus, which corresponds to greater stiffness. However, the addition of PBAT into the PLA matrix leads to a significant reduction in this modulus, attributed to the lower intrinsic stiffness of PBAT compared to PLA.^20,33^ This reduction becomes even more pronounced with the addition of essential oils, which further decrease the elastic modulus, albeit in a moderate manner. Nevertheless, this effect remains relatively stable even with increasing oil content.

Furthermore, both the maximum stress and strain at yield stress decrease with the addition of PBAT and essential oils, with the composite containing 10% cinnamon oil exhibiting the most significant reduction in these properties. This phenomenon occurs because essential oils can act as plasticizers, reducing tensile strength and increasing the plastic deformation capacity of the films. These findings align with previous studies that reported a decrease in the elastic modulus and tensile strength in polymeric matrices incorporated with eucalyptus and cinnamon oils.^26,37^ The decrease in elastic modulus observed with the incorporation of essential oils can be primarily attributed to their plasticizing effect, which enhances the mobility of polymer chains and reduces stiffness. Although poor interaction between the oils and the polymeric phases may also generate weak points, contributing to further loss of mechanical strength, the reduction in modulus was an expected outcome of oil addition.

Table 10 presents the mechanical properties of the systems in the plastic deformation region.

The results from Table 10 indicate that PLA, due to its inherent rigidity and brittleness, fractures at low deformations but under high stress levels, exhibiting a typical brittle material behavior.

The addition of PBAT significantly alters this mechanical response by increasing the material’s ductility, allowing greater elongation beyond the maximum stress point and promoting a transition to the plastic deformation region. This behavior is expected, given PBAT’s flexibility, which enhances plastic deformation capacity—an essential characteristic for biodegradable films. Both thyme and cinnamon oils reduce stress and toughness values, indicating interference with intermolecular interactions within the polymeric matrix. Among them, cinnamon oil exerts a more pronounced plasticizing effect, leading to a greater reduction in mechanical properties. In contrast, thyme oil, when combined with PBAT, exhibits a synergistic effect, promoting improved deformation behavior. Notably, composites containing thyme oil demonstrated higher elongation at rupture compared to PLA/PBAT and PLA/PBAT with cinnamon oil, suggesting that thyme oil contributes to a more balanced modification of mechanical performance.

Despite these variations in deformation and toughness, the stress levels in the plastic region remained relatively stable across all blends, indicating that the residual strength of the materials is not significantly affected by the presence of essential oils. A preview study conducted with PLA and PBAT blends and essential oils, like cinnamon, demonstrates that the interaction between the polymer matrices and the oils influence the mechanical properties as a result of the increase in the materials interaction, which increase the elongation behaviors by approximately five times comparing to blend without the oil, but on the other hand, it can also cause an increase in pore size of the film matrix, what leads to a debilitated structure and an amplified number of rupture points.^26^

Overall, the findings suggest that thyme oil provides a more favorable balance between stiffness and flexibility, reinforcing its potential for biodegradable plastic packaging applications. These results contribute to the understanding of how essential oils influence the mechanical behavior of PLA/PBAT-based compositions, supporting their strategic use in functional material development.

### Scanning Electron Microscopy (SEM)

The scanning electron microscopy (SEM) images from the fractured surfaces after cryogenic freezing are presented in Figures 6 and 7. Secondary electron (SE) detection was employed, as the primary objective of this analysis was to visualize the sample topography to better understand the compatibility between different biopolymers and the influence of essential oils on the interaction of these immiscible polymers with a third component in the blend.

**Figure 6:**
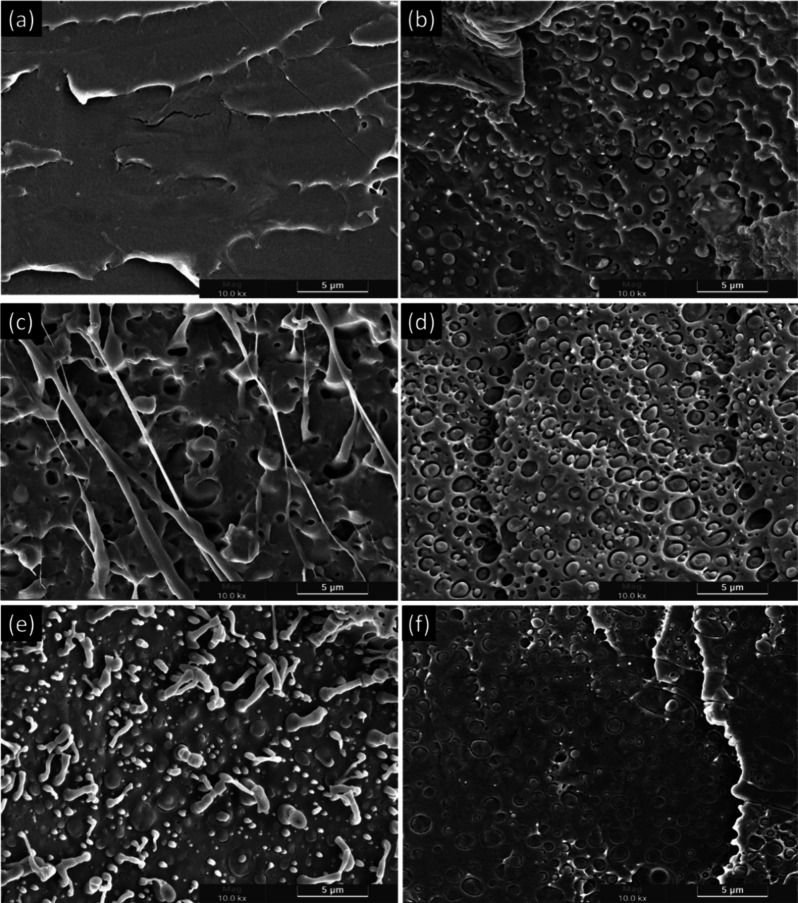
SEM images of injection-molded specimens of (a) PLA, (b) PLA/PBAT, (c) PLA/PBAT/T5, (d) PLA/PBAT/T10, (e) PLA/PBAT/C5, and (f) PLA/PBAT/C10, with a magnification of 10,000×.

**Figure 7:**
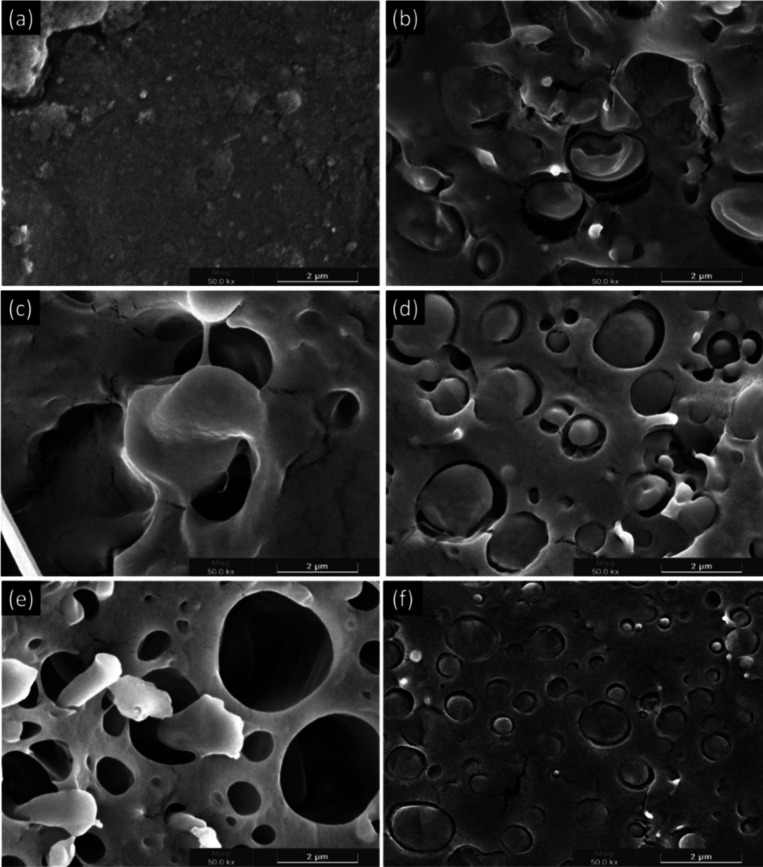
SEM images of injection-molded specimens of (a) PLA, (b) PLA/PBAT, (c) PLA/PBAT/T5, (d) PLA/PBAT/T10, (e) PLA/PBAT/C5, and (f) PLA/PBAT/C10, with a magnification of 50,000×.

PLA and PBAT are inherently immiscible biopolymers due to their low interfacial adhesion, which gives rise to a distinct two-phase morphology. This immiscibility stems from the disparity in intermolecular interactions between their polymer chains, as PLA exhibits strong dipole–dipole interactions due to its ester groups, whereas PBAT, with its aliphatic–aromatic structure, has a different polarity and chain flexibility. As a result, each polymer preferentially interacts with itself rather than forming stable interfacial interactions, leading to phase separation. SEM analysis corroborates this phenomenon, revealing well-defined PBAT domains dispersed within the continuous PLA matrix. The presence of interfacial voids at the PLA/PBAT boundaries further indicates the lack of adhesion, reinforcing the intrinsic incompatibility of the blend, as previously reported by Su et al. (2020).^38^

Interestingly, variations in the morphology of the dispersed PBAT phase were observed depending on processing conditions. In injection-molded specimens, PBAT domains appeared more elongated, suggesting that the shear and extensional forces during melt flow induced anisotropic deformation of the dispersed phase. This effect is commonly associated with polymer processing techniques that involve high shear rates, promoting the elongation of dispersed domains rather than their coalescence. However, despite this morphological transformation, no evidence of inherent compatibilization was identified in the binary system.

On the other hand, when essential oils were incorporated, differences in interfacial morphology became evident. In particular, the system containing 5 wt % thyme oil (Figure 7c) exhibited more continuous and less defined boundaries between PLA and PBAT, suggesting the presence of interfacial interaction zones. This may indicate a plasticizing effect that reduced the viscosity contrast between the phases, facilitating better dispersion and partial wetting at the interfaces. A similar trend, although less pronounced, could also be observed in the composition with 10 wt % cinnamon oil (Figure 7f), where localized regions of improved adhesion appeared. Nevertheless, the excess oil at higher concentrations may destabilize the system, promote the coalescence of dispersed domains and even generate interfacial defects. Therefore, the morphological evolution induced by essential oils seems to depend strongly on their concentration and chemical nature, with moderate additions favoring interfacial interaction, while excessive amounts compromise structural integrity.

## Conclusions

This study showed that the addition of thyme and cinnamon essential oils into PLA/PBAT blends effectively modified the properties of the resulting biosystems, highlighting their potential as functional additives in sustainable packaging. The two-step incorporation process allowed both essential oils to be integrated into the polymer blends. Although PLA and PBAT remained immiscible, as evidenced by the persistence of phase separation, the presence of thyme oil exhibited a synergistic effect with PBAT in enhancing the mechanical properties of PLA, increasing the elongation at break by approximately 100% compared to PLA/PBAT blends without oil.

Thermal analyses revealed that the essential oils improved the thermal stability of the blends and influenced their crystallization behavior. Rheological tests confirmed the reduction in viscosity and the predominance of viscous behavior in the samples.

Mechanical tests showed that while the addition of essential oils reduced the elastic modulus and tensile strength, it increased ductility—particularly with thyme oil—without severely compromising mechanical performance. SEM analyses confirmed the heterogeneous morphology of the blends, with phase separation between PLA and PBAT. However, in some compositions, such as PLA/PBAT with 5 wt % thyme oil, localized regions suggested improved wetting between phases, indicating that essential oils can modulate interfacial morphology without fully compatibilizing the system.

Furthermore, it is important to note that both thyme and cinnamon essential oils possess antimicrobial activity, as reported in the literature, adding further potential for their effective use in active packaging applications.

In conclusion, the results support the use of thyme and cinnamon essential oils as effective biobased modifiers to tailor the properties of PLA/PBAT composites for biodegradable and active packaging applications.

## Supplementary Material



## Notes

Pellets produced by extrusion (Figure S1); FTIR spectra of PLA and PBAT (Figure S2); DSC curves including second heating, cooling, and third heating scans (Figures S3–S5); and tensile stress–strain curves obtained from mechanical testing (Figure S6) (PDF)
