# Supplementary material for: Effect of Essential Oil Addition on PLA/PBAT Blend Properties for Biodegradable Packaging Applications
Source: ACS Omega. 2026 Mar 23;11(13):20320–33. doi: 10.1021/acsomega.5c10845 (PMC13063192; doi:10.1021/acsomega.5c10845)
Supplement: Supplementary file 1 [file ao5c10845_si_001.pdf]

EFFECT OF ESSENTIAL OIL ADDITION ON PLA/PBAT BLEND PROPERTIES FOR BIODEGRADABLE PACKAGING APPLICATIONS.

Murilo B. Valério[1], Ana Lúcia N. da Silva[1,2], Priscila S. e Souza[2], Marcelle M. Folena[3], Eduardo La M. da Silva[3].*

[1] Programa de Engenharia Ambiental, Escola Politécnica (PEA), Universidade Federal do Rio de Janeiro (UFRJ), Brasil, Rio de Janeiro, RJ.

[2] Instituto de Macromoléculas Professora Eloisa Mano (IMA), Universidade Federal do Rio de Janeiro (UFRJ), Brasil, Rio de Janeiro, RJ.

[3] Escola de Química, Universidade Federal do Rio de Janeiro (UFRJ), Brasil, Rio de Janeiro, RJ.

Keywords: PLA, PBAT, essential oils, thyme oil, cinnamon oil, biodegradable composites, active packaging.

Supporting information.

Figure S1. Image of pellets produced by extrusion: (a) PLA, (b) PLA/PBAT, (c) PLA/PBAT/T5, (d) PLA/PBAT/T10, (e) PLA/PBAT/C5, (f) PLA/PBAT/C10.

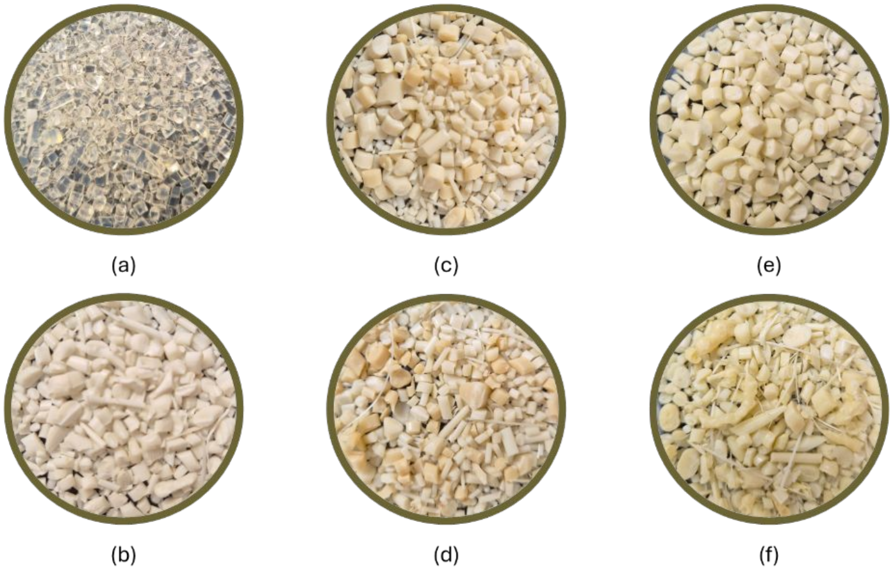

Figure S2. FTIR curves for PLA and PBAT.

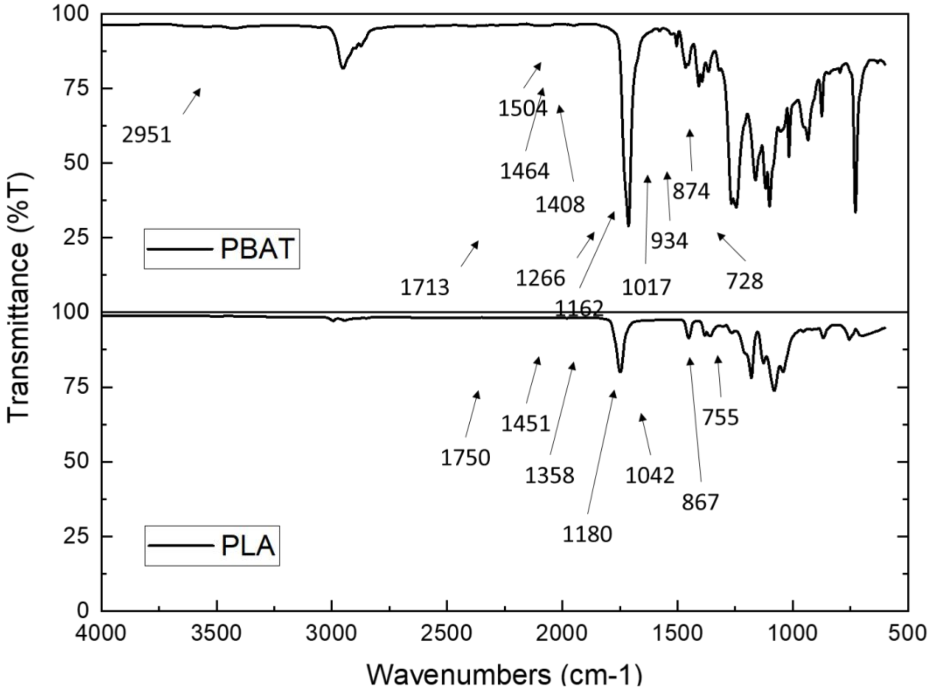

Figure S3. Second heat DSC curves.

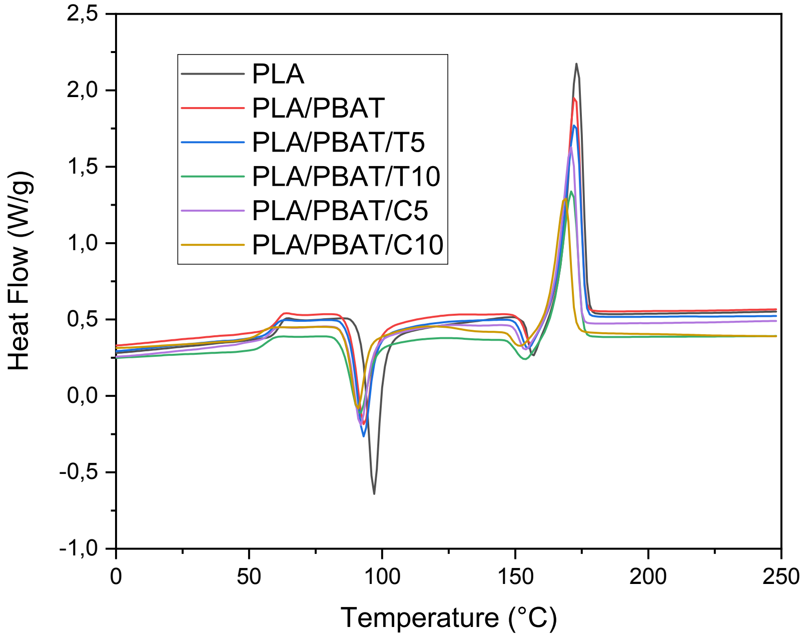

Figure S4. Cooling DSC curves.

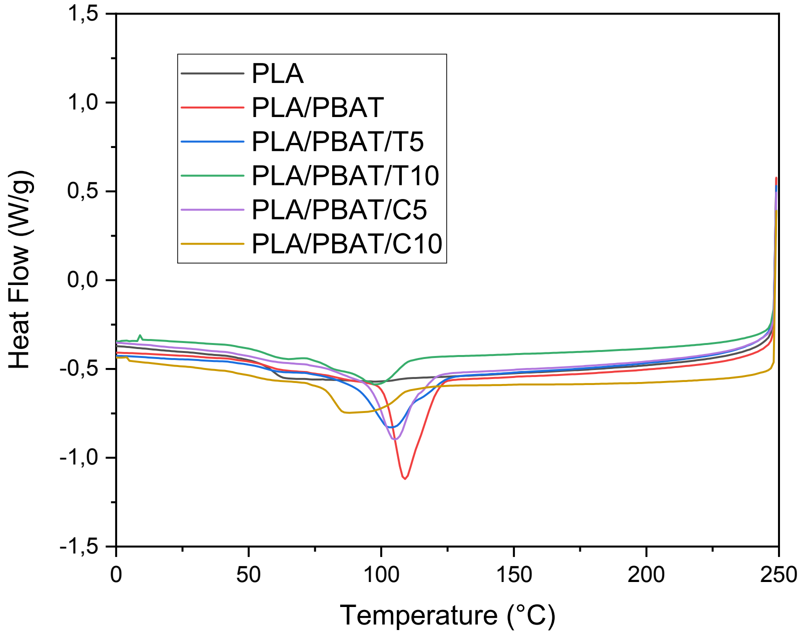

Figure S5. Third heat DSC curves.

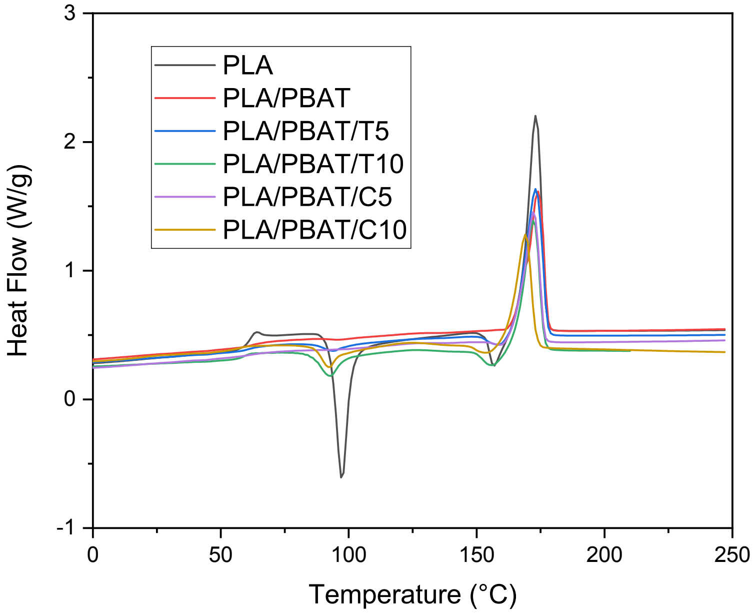

Figure S6. Mechanical responses curves obtained from tensile testing.

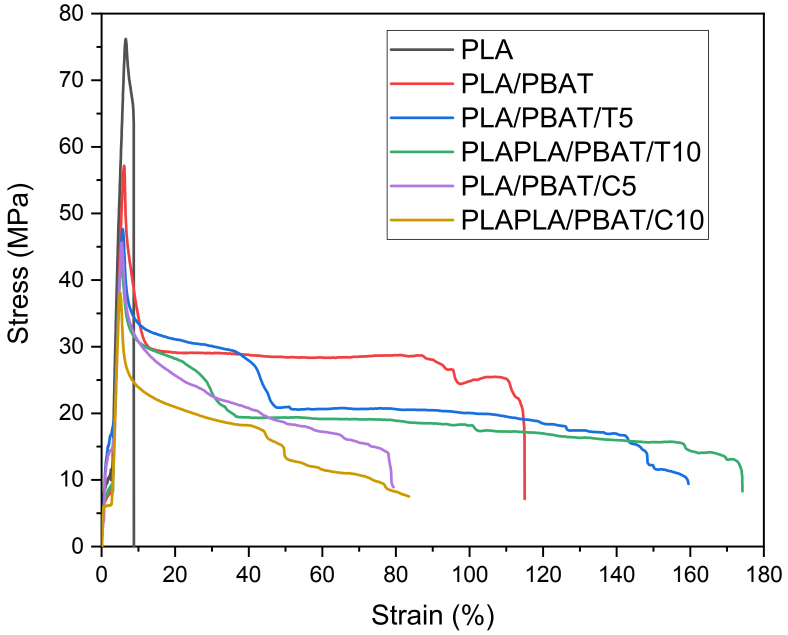
PLA
PLA/PBAT
PLA/PBAT/T5
PLAPLA/PBAT/T10
PLA/PBAT/C5
PLAPLA/PBAT/C10
Stress (MPa)
Strain (%)
0
10
20
30
40
50
60
70
80
0
20
40
60
80
100
120
140
160
180
